# Lifibrate attenuates blood-brain barrier damage following ischemic stroke via the MLCK/p-MLC/ZO-1 axis

**DOI:** 10.18632/aging.205692

**Published:** 2024-03-27

**Authors:** Yu Duan, Yao Deng, Feng Tang, Jian Li

**Affiliations:** 1 Department of Neurosurgery, Huadong Hospital Affiliated to Fudan University, Jing’an, Shanghai 200040, China

**Keywords:** Lifibrate, stroke, blood-brain barrier, tight junction proteins, ZO-1

## Abstract

Dysfunction of tight junction proteins-associated damage to the blood-brain barrier (BBB) plays an important role in the pathogenesis of ischemic stroke. Lifibrate, an inhibitor of cholinephosphotransferase (CPT), has been used as an agent for serum lipid lowering. However, the protective effects of Lifibrate in ischemic stroke and the underlying mechanism have not been clearly elucidated. Here, we employed an *in vivo* mice model of MCAO and an OGD/R model *in vitro*. In the mice models, neurological deficit scores and infarct volume were assessed. Evans Blue solution was used to detect the BBB permeability. The TEER was examined to determine brain endothelial monolayer permeability. Here, we found that Lifibrate improved neurological dysfunction in stroke. Additionally, increased BBB permeability during stroke was significantly ameliorated by Lifibrate. Correspondingly, the reduced expression of the tight junction protein ZO-1 was restored by Lifibrate at both the mRNA and protein levels. Using an *in vitro* model, we found that Lifibrate ameliorated OGD/R-induced injury in human bEnd.3 brain microvascular endothelial cells by increasing cell viability but reducing the release of LDH. Importantly, Lifibrate suppressed the increase in endothelial monolayer permeability and the reduction in TEER induced by OGD/R via the rescue of ZO-1 expression. Mechanistically, Lifibrate blocked activation of the MLCK/ p-MLC signaling pathway in OGD/R-stimulated bEnd.3 cells. In contrast, overexpression of MLCK abolished the protective effects of Lifibrate in endothelial monolayer permeability, TEER, as well as the expression of ZO-1. Our results provide a basis for further investigation into the neuroprotective mechanism of Lifibrate during stroke.

## INTRODUCTION

Ischemic stroke (IS) is a disease characterized by narrowing or occlusion of the middle cerebral or vertebrobasilar arteries, resulting in insufficient blood supply to certain parts of the brain and causing cerebral infarction, accompanied by symptoms including hemiplegia and disturbance of consciousness which may lead to a series of complications [1]. The incidence of IS increases year by year, and it has become the second leading cause of human death and the third leading cause of disability [2]. Currently, the main drug used to treat IS in clinical practice is recombinant tissue plasminogen activator (tPA), which is also the only drug approved by the Food and Drug Administration (FDA) for its treatment [3]. However, the narrow treatment window and potential for causing hemorrhagic transformation greatly limit the clinical application of tPA [4]. In recent years, research on IS has made progress. It has been shown that inhibiting neuronal apoptosis in the pathological process of IS can improve cerebral ischemia-hypoxia and subsequent neurologic damage caused by perfusion [5]. However, there is no effective therapeutic drug with this specific mechanism. It is evident that further clarification of the pathogenesis of IS-induced neuronal injury, identification of therapeutic targets, and development of therapeutic drugs are extremely urgent scientific tasks.

It is well known that one characteristic of IS is injury to the blood-brain barrier (BBB), leading to damage to ionic homeostasis and brain transporter function [6, 7]. The mechanism of BBB injury caused by IS includes changes to tight junction (TJ) proteins such as occludin, claudin-1, and ZO-1, regulation of transporter expression, and inflammatory damage. Structural damage to TJ proteins combined with dysfunction of BBB transporter proteins can jointly increase paracellular solute permeability, ultimately leading to cerebral edema and exacerbating brain damage [8]. ZO-1 is a membrane-associated guanylate kinase-like protein, linking transmembrane tight junction proteins to the actin cytoskeleton. Dissociation of ZO-1 from the junction complex causes increased BBB permeability, indicating that the ZO-1-transmembrane protein interaction is critical to tight junction stability and function [9, 10]. Numerous studies have shown that BBB injury increases neuronal damage and cerebral inflammation caused by IS, playing a vital role in the pathological response to IS [11–14]. The BBB is formed by the interaction between endothelial cells and microvessels, astrocytes, microglia, pericytes, neurons, and extracellular matrix [15, 16]. The BBB expresses multiple ion transporters and channels that regulate ion concentrations in the central nervous system, maintaining a stable extracellular environment for the brain parenchyma [17]. In the absence of a functional BBB, this ion concentration fluctuation may lead to serious interference in neuronal and glial signaling [18]. Protecting the BBB from damage after IS is crucial to reducing cerebral injury.

Lifibrate (1-Methyl-4-piperidinyl bis (4-chloro-phenoxy) acetate), also named SAH 42-348, is an inhibitor of cholinephosphotransferase (CPT), which is applied for the clinical treatment of hyperlipidemia with promising outputs [19]. However, the potential role of Lifibrate in treating IS remains uncertain. Herein, the *in vivo* and *in vitro* protective function of Lifibrate against ischemia-induced BBB damage was evaluated to explore its possible capacity in the clinical treatment of IS.

## MATERIALS AND METHODS

### Experimental model and drug administration

Forty C57BL/6J male mice were obtained from Charles River (Beijing, China) and divided into 3 groups: (1) Vehicle; (2) MCAO; (3) Lifibrate+MCAO. In the Lifibrate+MCAO group, mice were orally administered with 35 mg/kg/day Lifibrate (Cat#T27831, Targetmol, USA) for 7 days before the MCAO experiments. In the vehicle and MCAO groups, animals were orally dosed with the same volume of vehicle solution for 7 days prior to the Sham and MCAO operation, respectively. For the MCAO modeling, the mouse was anesthetized with a 10% chloral hydrate (0.35 g/kg) intraperitoneal injection and then fixed in the supine position after the anesthesia took effect. The mouse’s neck was prepared and disinfected, and a 1.0 cm incision was made at its center. A stereoscopic microscope was used to aid the procedure of separating the right and external carotid arteries, followed by ligating the external carotid artery with a suture and making a small incision. A nylon embolus was inserted into the carotid artery through this incision, and the insertion was stopped when resistance was felt. Then the embolus was fixed, which blocked the middle cerebral artery of the mouse. After one hour of ischemia, the nylon embolus was removed promptly, and perfusion was performed. Lastly, the incision was closed, and the MCAO model was complete. For the Sham operation group, after preparation and disinfection, the mouse’s neck skin was incised, and only the right and external carotid arteries were exposed and separated. When the blood flow decreased by 70-80%, the model was successfully established. During the surgery, the mouse’s body temperature was maintained and vital signs were observed. After the surgery, the mouse was transferred to a 37° C incubator. After waking up, the mouse was given appropriate food and water.

### 2,3,5-triphenyl tetrazolium chloride (TTC) staining

The TTC staining was performed according to the previous study [20]. After anesthesia, the head skin of the mouse was cut open, the skull opened, and the brain tissue removed. The olfactory bulb, cerebellum, and brainstem were removed, and 5 consecutive slices were made, each slice thickness was 2 mm from front to back. The brain slices were placed in TTC solution (2% concentration, Cat#G3005, Solarbio, Beijing, China) and incubated in the dark for 15 min and turned over every 7-8 minutes. After successful staining of the brain slices, normal brain tissue was stained red and the infarcted tissue stained white. After completing the photography of the brain slices, the area of cerebral infarction was calculated using Image-Pro Plus 6.0 software. The formula was shown as follows: cerebral infarction area = (white infarct area/total brain slice area) × 100%.

### Neurological deficit scoring

The neurological deficit of each mouse was evaluated using the Longa scoring method described previously [21]. The neurologic findings were scored on a five-point scale. 0 indicated no neurologic deficit; 1 indicated mild focal neurologic deficit, characterized by failure to fully extend the left or right forepaw; 2 indicated moderate focal neurologic deficit, manifested by circling to the left; 3 indicated severe focal neurologic deficit characterized by falling to the left; 4: indicated a depressed level of consciousness and lack of spontaneous walking.

### Blood-brain barrier permeability detection

After intravenous injection of 2% Evans Blue solution (1 mL) through the tail vein, the eyes and skin of the mice turned blue within a few seconds. One hour later, physiological saline was perfused through the heart to wash out the Evans Blue solution. The ischemic hemisphere was incubated with dimethylformamide at 60° C for 24 hours. The concentration of Evans Blue was detected using spectrophotometry at a wavelength of 620 nm to evaluate BBB leakage [22].

### Immunostaining of ZO-1

The expression of ZO-1 was detected using immunofluorescence staining as described before [23]. The mouse brain tissue was fixed with 4% paraformaldehyde and dehydrated with 30% sucrose, followed by being embedded and sliced into sections (20 μm). The sections were incubated with blocking solution (5% horse serum and 0.1% Triton X-100) for 90 min, and then incubated with ZO-1 primary antibody (1:100, Cat#ab307799, Abcam, USA) at 4° C overnight. After adding the corresponding fluorescent secondary antibody (1:200, Abcam, USA), the sections were incubated for 90 min. The slides were sealed with nail polish and air-dried in a Fume Hood before being photographed using a fluorescence microscope (Leica, Wetzlar, Germany).

### Cell culture and treatments

The mouse brain microvascular endothelial cells (bEnd.3 cells) were obtained from ATCC (USA) and cultured in high-glucose endothelial cell medium (ECM) containing 10% FBS and then cultured under 5% CO_2_ at 37° C. For the construction of the OGD/R model [24], logarithmic-phase bEnd.3 cells were digested and seeded into culture dishes. After cells adhered to the dish, the medium was replaced with a glucose-free medium, and the culture dish was placed in a hypoxia chamber (95% N_2_, 5% CO_2_) in a pre-adjusted culture box at 37° C for oxygen-glucose deprivation culture for 4 h. Afterwards, the glucose-free medium was discarded, and cells were cultured in a complete medium under normoxic conditions (37° C, 5% CO_2_) for 24 h. To construct the MLCK-overexpressed bEnd.3 cells, cells were transfected with the lentivirus containing the MLCK-overexpressed vector (lentiviral-MLCK) for 48 h, which was identified using the Western blotting assay.

### 3-(4,5-dimethylthiazol-2-yl)-2,5-diphenyltetrazolium bromide (MTT) assay

After completion of the indicated treatment, 20 μl of MTT solution (Cat#ab211091, Abcam, USA) was loaded for 4-h culture, followed by loading 200 μl DMSO and shaking. The absorbance (OD) value was detected at 490 nm using a microplate reader (MD, USA).

### Lactate dehydrogenase (LDH) release

Cells in the logarithmic growth phase were seeded in a 96-well culture plate, and the cell culture medium of each group was collected in 1.5 mL centrifuge tubes. LDH activity was detected according to the instructions on the LDH kit (Mibio, Shanghai, China).

### Trans-endothelial electrical resistance (TEER)

Cells were seeded into a 12-well Transwell chamber and cultured as a monolayer. The TEER value was measured at 4, 8, 12, 16, 20, 24, 48, 72, and 96 hours after seeding. After the measured resistance was stable, different groups were established. The culture media in both the inside and outside of the Transwell chamber were discarded, and completely replaced with fresh media, maintaining the same liquid level inside and outside. After the modeling and drug treatments, TEER was measured. The calibrated Millicell-ERS-2 electrode was immersed in 70% ethanol for 15 min and then dried in the air for 5 s. The electrode was washed with a sterile electrolyte solution. The short and long ends of the electrode were immersed in the culture media inside and outside the Transwell chamber, maintaining perpendicularity with the liquid surface. Finally, data were read and recorded.

### The detection of endothelial permeability

1 mg FITC Dextran (Cat#T18988, Targetmol, USA) was dissolved in 1 ml double-distilled water to obtain a stock solution with a concentration of 1 mg/ml. The stock solution was diluted with serum-free medium to a working concentration of 500 μg/ml. After this intervention, PBS was added for washing twice. 100 μl FITC Dextran (500 μg/ml) was added to the upper compartment, and 600 μl serum-free basal medium was added to the lower compartment. After 1 h incubation in the dark, 100 μl medium was aspirated from each compartment and loaded onto a black 96-well plate. The OD value was measured at 495nm/520nm using a multi-functional enzyme reader (MD, USA). The permeability was calculated [25].

### Real-time PCR assay

RNA was extracted following the protocol provided with the Takara RNA extraction kit (Takara, Kusatsu, Japan). The concentration and purity of the total RNA were determined using a OneDrop micro-ultraviolet spectrophotometer. cDNA was synthesized from mRNA using the protocol provided with the Takara PrimeScript RT-PCR kit (Takara, Japan). Quantitative Real-time PCR (qPCR) was performed according to the protocol provided with the Takara Premix Ex TaqTM kit (Takara, Japan). The relative expression levels of genes were analyzed using the 2^-ΔΔCT^ method, with the housekeeping gene GAPDH as the reference.

### Western blotting assay

Cellular samples were collected and lysed, and the protein concentration was determined using the BCA method. Based on the target protein molecular weight, a suitable concentration of protein electrophoresis gel was selected for protein electrophoresis, membrane transfer, and then blocked for 1 h. Primary antibodies against ZO-1 (1:1000, Cat#AF5145, Affinity Biosciences, China), MLCK (1:800, Cat#AF5314, Affinity Biosciences, China), p-MLC (1:1000, Cat#95777, Cell Signaling Technology, USA), MLC (1:1000, Cat#8505, Cell Signaling Technology, USA), and β-actin (1:2000, Cat#4970, Cell Signaling Technology, USA) were incubated overnight at 4° C on a shaker. Secondary antibodies (1:5000, Cat#58802, Cell Signaling Technology, USA) were then incubated for exposure, and the images were recorded using a Bio-Rad Chemidoc XRS+ imaging system. The protein expression levels were calculated using the software ImageJ for grayscale analysis.

### Statistical analysis

Statistical analysis was performed using the software GraphPad Prism 8.3. Quantitative data were presented as mean ± standard deviation. Comparisons between groups were conducted using one-way analysis of variance. The Bonferroni test was used as a post hoc test. *P*<0.05 was taken as statistically significant.

### Data availability

All data for this study are available on reasonable request from the corresponding author.

## RESULTS

### Lifibrate decreased brain infarction volume and improved neurological dysfunction in a MCAO mice model

**Figure 1:**
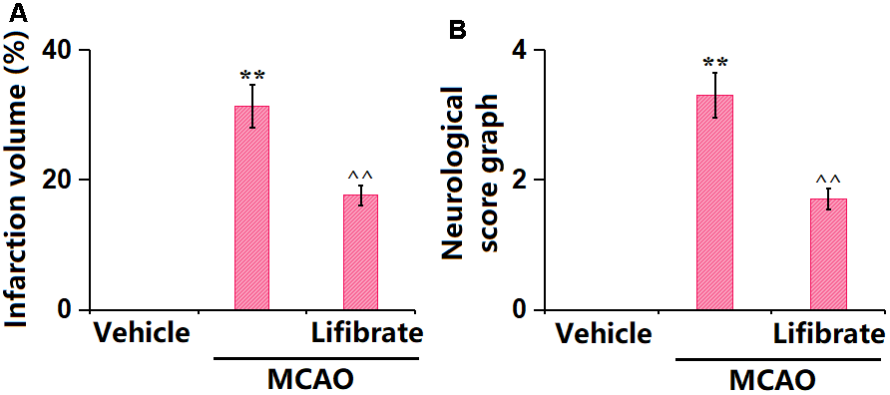
**Lifibrate decreased brain infarction volume and improved neurological dysfunction in a middle cerebral artery occlusion (MCAO) mice model.** Mice were divided into 3 groups: (1) Vehicle; (2) MCAO; (3) Lifibrate+MCAO. (**A**) Quantification of infarction volume; (**B**) Neurological score graph of the three experimental groups (n=10, **, P<0.01 vs. vehicle group; ^^, P<0.01 vs. MCAO group).

In this study, we aimed to explore the pathological changes in MCAO mice. Animals were orally administered with 35 mg/kg/day Lifibrate for 7 days before MCAO modeling. The percentage of infarction volume in MCAO mice was markedly increased to 31.3%±3.25%, which was significantly reduced to 17.6%±1.56% by 35 mg/kg Lifibrate (Figure 1A). Furthermore, the neurological deficit scores in the vehicle, MCAO, and Lifibrate+MCAO groups were 0, 3.3±0.35, and 1.7±0.16, respectively (Figure 1B).

### Lifibrate ameliorated aggravation of BBB permeability in a MCAO mice model

**Figure 2:**
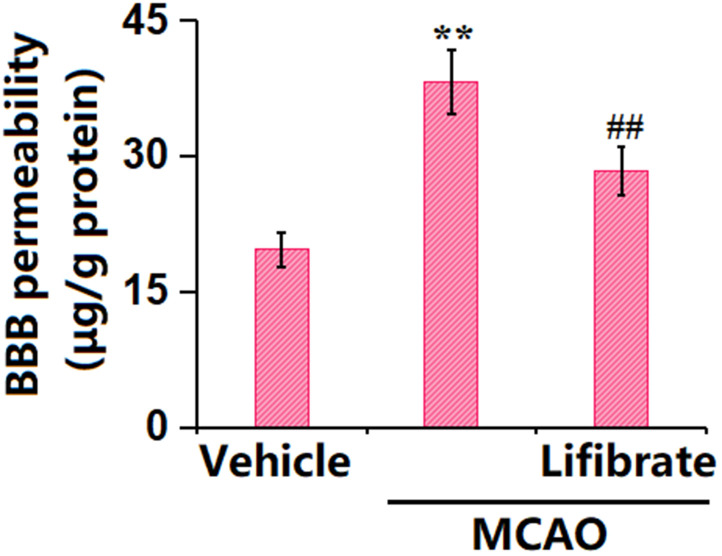
**Lifibrate ameliorated aggravation in blood-brain barrier (BBB) permeability in a middle cerebral artery occlusion (MCAO) mice model.** Blood-brain barrier permeability was assayed by Evans blue staining (n=10, **, P<0.01 vs. vehicle group; ^^, P<0.01 vs. MCAO group).

Evans blue staining is the most common way to assess BBB leakage [26]. Here, the dye Evans blue was injected into the mice through their tails. The concentration of Evans blue in MCAO mice was significantly increased from 19.6±1.82 to 38.1±3.56 μg/g protein, which was markedly reduced to 28.3±2.61 μg/g protein by 35 mg/kg Lifibrate (Figure 2), revealing a protective function of Lifibrate against BBB dysfunction in MCAO mice.

### Lifibrate restored expression of the TJ protein ZO-1 in a MCAO mice model

**Figure 3:**
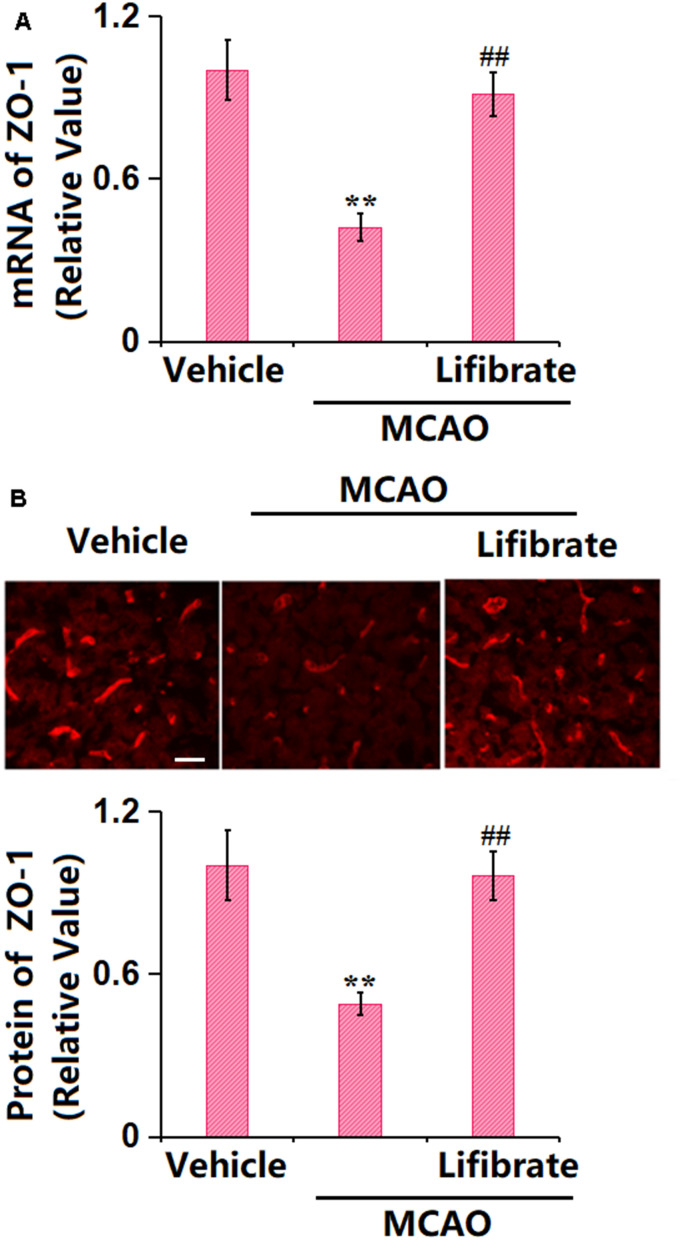
**Lifibrate restored expression of the tight junction protein ZO-1 in a middle cerebral artery occlusion (MCAO) mice model.** (**A**) mRNA of ZO-1; (**B**) Immunostaining of ZO-1. Scale bar, 100 μm (n=10, **, P<0.01 vs. vehicle group; ^^, P<0.01 vs. MCAO group).

TJ proteins are critical components of the BBB [27]. In MCAO mice, the gene (Figure 3A) and protein (Figure 3B) levels of ZO-1 were found notably decreased, but sharply elevated by 35 mg/kg Lifibrate, implying a repairment role of Lifibrate on the TJ structure in MCAO mice.

### Lifibrate mitigated OGD/R-triggered insults in human bEnd.3 cells

**Figure 4:**
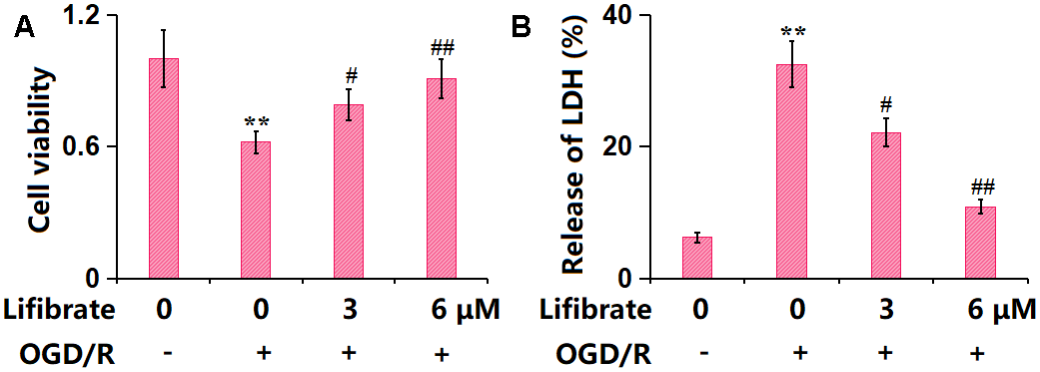
**Lifibrate mitigated oxygen-glucose deprivation/reperfusion (OGD/R)- induced insults in bEnd.3 brain microvascular endothelial cells.** Cells were exposed to hypoxic conditions for 6 h, followed by exposure to reperfusion media for 24 h in the presence or absence of Lifibrate (3, 6 μM). (**A**) Cell viability was determined by MTT assay; (**B**) Release of LDH (n=10, **, P<0.01 vs. vehicle group; #, ##, P<0.05, 0.01 vs. OGD/R group).

To explore the possible functional mechanism, bEnd.3 cells were exposed to the OGD/R condition with or without Lifibrate (3, 6 μM). The cell viability was reduced from 100% to 62±0.05% in OGD/R-handled cells, then promoted to 79±0.07% and 91±0.09% by 3 and 6 μM Lifibrate, respectively (Figure 4A). Moreover, the LDH release values in the control, OGD/R, 3 μM Lifibrate, and 6 μM Lifibrate groups were 6.2±0.72%, 32.5±3.51%, 22.1±2.13%, and 10.9±1.05%, respectively (Figure 4B).

### Lifibrate attenuated OGD/R-triggered aggravation in brain endothelial monolayer permeability in bEnd.3 cells

**Figure 5:**
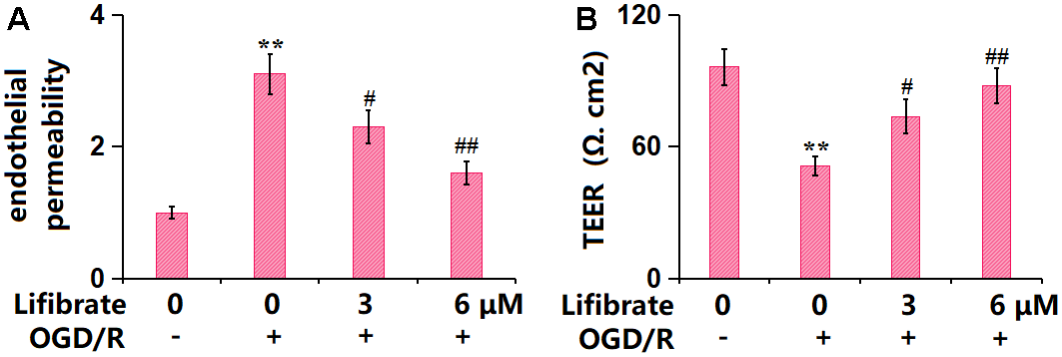
**Lifibrate attenuated OGD/R-induced aggravation in brain endothelial monolayer permeability in bEnd.3 brain microvascular endothelial cells.** Cells were exposed to hypoxic conditions for 6 h, followed by exposure to reperfusion media for 24 h in the presence or absence of Lifibrate (3, 6 μM). (**A**) Brain endothelial permeability was assessed by FITC-dextran permeation; (**B**) TEER on the endothelial monolayer was measured (n=6, **, P<0.01 vs. vehicle group; #, ##, P<0.05, 0.01 vs. OGD/R group).

The endothelial monolayer permeability of bEnd.3 cells was assessed by FITC-dextran permeation and TEER detection. The increased concentration of FITC-dextran observed in OGD/R-challenged cells was remarkably repressed by 3 and 6 μM Lifibrate (Figure 5A). In addition, the TEER value was declined from 96.2±8.35 to 51.3±4.21 Ω. cm^2^ in OGD/R-treated cells, then promoted to 73.7±7.86 and 87.9±8.02 Ω. cm^2^ by 3 and 6 μM Lifibrate, respectively (Figure 5B).

### Lifibrate preserved the expression of the TJ protein ZO-1 against OGD/R in bEnd.6 cells

**Figure 6:**
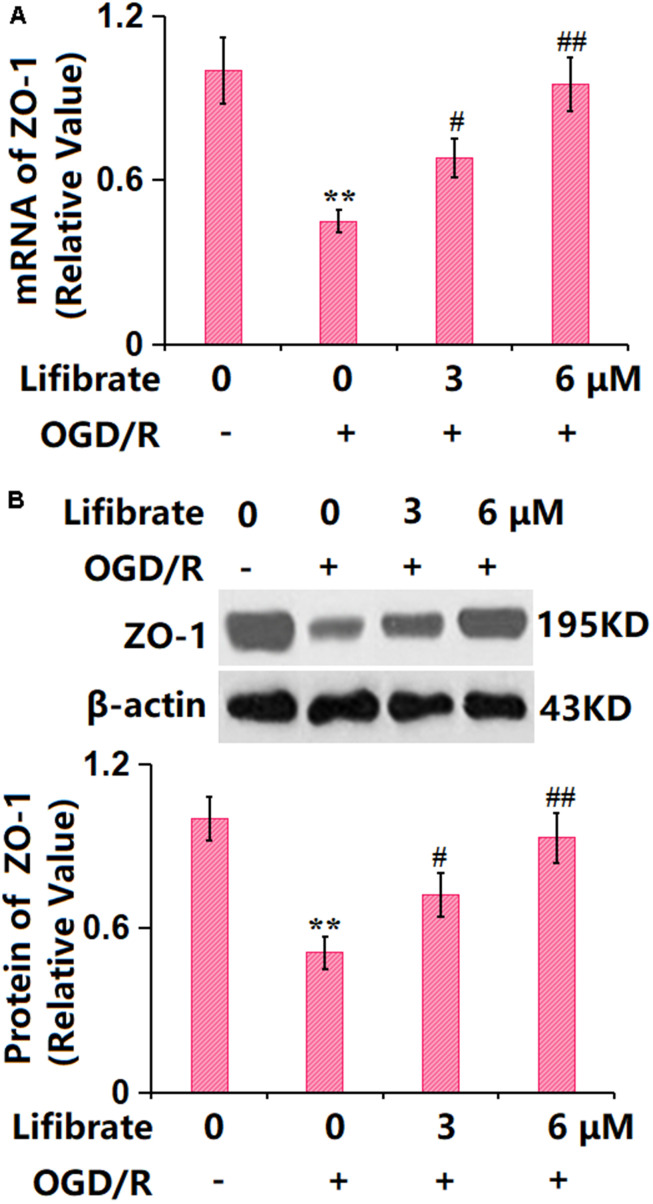
**Lifibrate reserved expression of the tight junction protein ZO-1 against OGD/R in bEnd.6 cells.** Cells were exposed to hypoxic conditions for 6 h, followed by exposure to reperfusion media for 24 h in the presence or absence of Lifibrate (3, 6 μM). (**A**) mRNA of ZO-1; (**B**) Protein expression of ZO-1 (n=6, **, P<0.01 vs. vehicle group; #, ##, P<0.05, 0.01 vs. OGD/R group).

In line with the *in vivo* data, ZO-1 was found sharply downregulated in OGD/R-treated cells, but remarkably upregulated by 3 and 6 μM Lifibrate (Figure 6A, 6B), confirming the protection of TJ structure by Lifibrate.

### Lifibrate inhibited the activation of MLCK/p-MLC signaling against OGD/R in bEnd.3 cells

**Figure 7:**
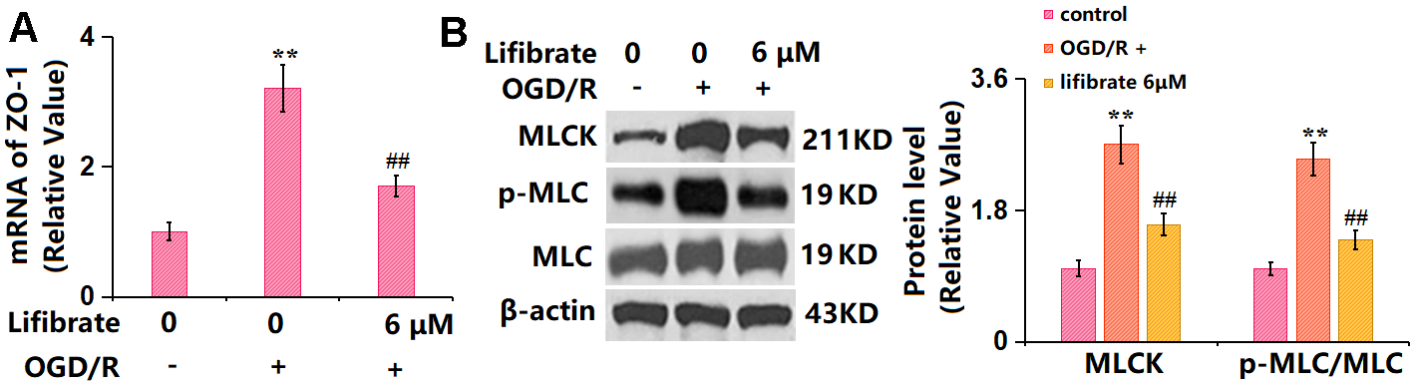
**Lifibrate inhibited activation of the MLCK/p-MLC signaling against OGD/R.** Cells were exposed to hypoxic conditions for 6 h, followed by exposure to reperfusion media for 24 h in the presence or absence of Lifibrate (6 μM). (**A**) mRNA of MLCK; (**B**) Protein levels of MLCK and p-MLC/MLC (n=6, **, P<0.01 vs. vehicle group; ##, P< 0.01 vs. OGD/R group).

MLCK/p-MLC signaling is claimed as a critical pathway that mediates the expression of TJ proteins [28]. bEnd.3 cells were exposed to the OGD/R condition with or without Lifibrate (6 μM). The gene (Figure 7A) and protein (Figure 7B) levels of MLCK were notably increased in OGD/R-challenged cells but sharply suppressed by 6 μM Lifibrate. Furthermore, the markedly increased p-MLC/MLC level observed in OGD/R-handled cells was remarkably repressed by 6 μM Lifibrate (Figure 7B).

### Overexpression of MLCK abolished the beneficial effects of Lifibrate in ZO-1 expression and endothelial permeability

**Figure 8:**
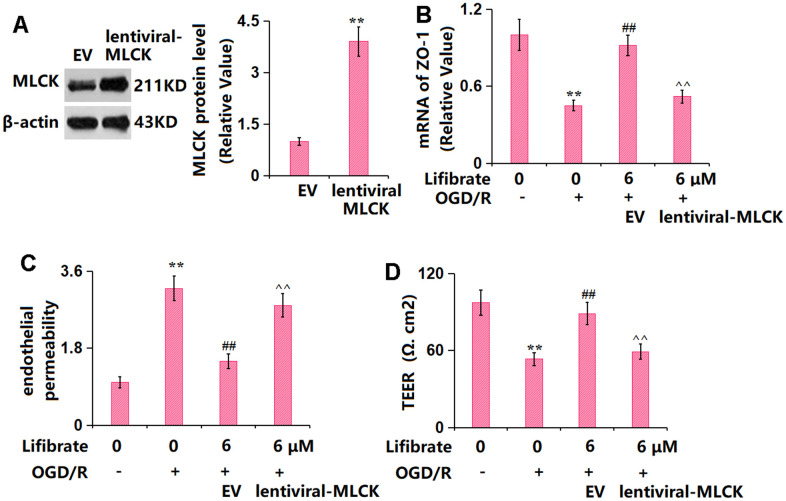
**Overexpression of MLCK abolished the beneficial effects of Lifibrate in ZO-1 and endothelial permeability.** Cells were transducted with lentiviral-MLCK, followed by exposure to OGD/R with or without Lifibrate (6 μM). (**A**) Successful overexpression of MLCK was detected by Western blot analysis; (**B**) mRNA of ZO-1; (**C**) Endothelial permeability; (**D**) TEER on the endothelial monolayer (n=6, **, P<0.01 vs. vehicle group; ##, P<0.01 vs. OGD/R group; ^^, P<0.01 vs. OGD/R+ Lifibrate group).

To identify the role of MLCK/p-MLC signaling in the function of Lifibrate, cells were transduced with lentiviral-MLCK, followed by exposure to OGD/R with or without Lifibrate (6 μM). The successful overexpression of MLCK in bEnd.6 cells was verified by the Western blotting data (Figure 8A). The markedly declined ZO-1 level in OGD/R-treated cells was significantly elevated by Lifibrate, which was remarkably reversed by MLCK overexpression (Figure 8B). Moreover, the increased concentration of FITC-dextran OGD/R-handled cells was notably repressed by Lifibrate and then observably elevated by MLCK overexpression (Figure 8C). Furthermore, the TEER value was declined from 97.1±9.82 to 53.3±5.22 Ω. cm^2^ in OGD/R-handled cells but significantly increased to 88.8±8.82 Ω. cm^2^ by Lifibrate. After overexpressing MLCK, the TEER value was markedly decreased to 59.3±6.11 Ω. cm^2^ (Figure 8D).

## DISCUSSION

In this study, using an *in vivo* MCAO model and an *in vitro* OGD/R model, we demonstrated a novel pharmacological function of Lifibrate by showing that its administration could ameliorate ischemia-induced BBB damage. We also found that Lifibrate reversed the pathological changes, suppressed the BBB leakage and restored the expression of TJ-related proteins both *in vivo* and in *vitro*. Furthermore, we proved that these effects are mediated by MLCK/p-MLC signaling. As early as the beginning of the 20th century, the concept of the BBB was proposed by Lewandowsky et al. Subsequent research by Goldman discovered dyes did not enter the brain from the blood but rather penetrated via cerebrospinal fluid (CSF) into the brain, further strengthening the concept of the BBB [29–31]. When endothelial cells form the vessel wall, pericytes are embedded in the basement membrane, while astrocytes completely envelop cerebral capillaries [32]. Although endothelial cells and their TJ proteins constitute the ultimate permeability barrier, pericytes, and astrocytes also play important regulatory roles. The BBB acts as a crosstalk site between multiple central nervous system cells and peripheral cells and plays a fundamental role in maintaining homeostasis and normal neuronal function within the central nervous system [33, 34]. Cerebral edema caused by IS includes cytotoxic and vasogenic edema. Cerebral edema gradually worsens after ischemia, with cytotoxic edema developing minutes after ischemia and vasogenic edema appearing relatively late, particularly in association with BBB breakdown [35]. In patients with IS, BBB breakdown allows a large amount of blood-derived fluid to enter the brain parenchyma, ultimately leading to progressive increases in water content within brain tissue [36]. Under physiological conditions, cerebral endothelial cells are connected and fused through TJ proteins and adherens junction proteins. The tight junction proteins include Occludin and Claudin-5, while the adherens junction protein VE-Cadherin is anchored to the actin cytoskeleton by Zonula Occludins (ZO). The dynamic interaction between the cytoskeleton and junction proteins is crucial for maintaining BBB function. Upon exposure to specific stressors, actin polymerization occurs in normal situations, which is accompanied by actinomyosin contraction and elevated cytoskeletal tension, leading to cell shape contraction and damage to the barrier formed by junction proteins. Meanwhile, the loss of TJ proteins further increases the gap between endothelial cells, resulting in increased BBB permeability in IS [37, 38]. Herein, in line with previous data presented by Song [39], increased infarct size and neurological deficit score were observed in the MCAO mouse model, both of which were significantly alleviated by 35 mg/kg Lifibrate, implying its protective role against IS. Furthermore, consistent with the results presented by Huang [40] and Cao [41], increased BBB permeability and endothelial permeability were observed in MCAO mice and OGD/R-treated bEnd.3 cells, respectively. Following the administration of Lifibrate, the BBB permeability in MCAO mice and endothelial permeability in OGD/R-handled bEnd.3 cells were significantly repaired, suggesting that the function of Lifibrate in IS might be connected to the BBB repairment.

ZO-1 is a crucial structural protein in the TJ complex and the first TJ adhesive protein to be identified [42]. It connects to transmembrane proteins (occludin, Claudin, etc.) on one hand and to cytoskeletal proteins on the other. Changes in the expression levels of ZO-1 are correlated with the degree of BBB damage, making it a valuable assessment indicator for blood-brain barrier damage and function [43]. Herein, as mentioned by Zeng et al. [44], the ZO-1 content was remarkably declined in both MCAO mice and OGD/R-challenged bEnd.3 brain endothelial cells and largely restored by Lifibrate, indicating that the protection of Lifibrate against IS might be correlated to ZO-1-mediated BBB repairment. Myosin light chain (MLC) is divided into a basic light chain and a regulatory light chain. The former stabilizes the structure of the heavy chain, while the latter participates in regulating the activity of myosin, which is a molecular motor of the cell cytoskeleton and participates in various physiological activities of cells [45]. Phosphorylation of MLC (p-MLC) is mainly regulated by the dual modulation of the myosin light chain kinase (MLCK) system and the Rho kinase system, which together regulate the dynamic balance between non-phosphorylation and phosphorylation of MLC [46]. p-MLC is increased when MLCK is activated by upstream calcium-calmodulin complexes. Rho kinases are activated by the activation signal transmitted by Rho, phosphorylating MLC phosphatase (MLCP) in BBB endothelial cells and inactivating it. The dephosphorylation of MLC cannot be induced by inactivated MLCP, leading to an increase in p-MLC expression and endothelial cell permeability [47]. Herein, the MLCK/p-MLC signaling in OGD/R-handled bEnd.3 cells was markedly activated, in line with Li’s report [48]. After introducing Lifibrate, the activity of the MLCK/p-MLC pathway was repressed. Moreover, the impact of Lifibrate on the endothelial permeability and ZO-1 level in OGD/R-handled bEnd.3 cells was notably abolished by overexpressing MLCK, confirming that Lifibrate repaired the BBB permeability by upregulating ZO-1 via inactivating the MLCK/p-MLC signaling. In our future work, the interaction between Lifibrate and the Rho kinase system will be further studied to investigate the possibility of other mechanisms of action.

Taken together, Lifibrate attenuated BBB damage following IS via regulating the MLCK/p-MLC/ZO-1 axis. Our findings are helpful for improving the understanding of the intricacy of the mechanism of Lifibrate-ameliorated neurological impairment and highlighting its potential as an effective candidate for the treatment of ischemic insult in patients who are susceptible to stroke.
